# Desynchronized white matter function and structure in drug-naive first-episode major depressive disorder patients

**DOI:** 10.3389/fpsyt.2022.1082052

**Published:** 2023-01-11

**Authors:** Qinger Guo, Jingfeng Duan, Shuyang Cai, Jiaxi Zhang, Tao Chen, Hong Yang

**Affiliations:** ^1^Department of Radiology, The First Affiliated Hospital, College of Medicine, Zhejiang University, Hangzhou, China; ^2^Department of Psychiatry, The First Affiliated Hospital, College of Medicine, Zhejiang University, Hangzhou, China; ^3^School of Teacher Education, Zhejiang Normal University, Jinhua, China; ^4^Key Laboratory of Intelligent Education Technology and Application of Zhejiang, Zhejiang Normal University, Jinhua, China

**Keywords:** major depressive disorder, white matter, resting-state functional magnetic resonance imaging, amplitude of low frequency fluctuation, diffusion tensor imaging, tract-based spatial statistics, tractography

## Abstract

**Background:**

Major depressive disorder (MDD) is a highly prevalent mental disease. Using magnetic resonance imaging (MRI), although numerous studies have revealed the alterations in structure and function of grey matter (GM), few studies focused on the synchronization of white matter (WM) structure and function in MDD. The aim of this study was to investigate whether functional and structural abnormalities of WM play an essential role in the neurobiological mechanisms of MDD.

**Methods:**

Gradient-echo imaging sequences at 3.0T were used to gather resting state functional MRI (rsfMRI) data, which were performed on 33 drug-naive first-episode MDD patients and 34 healthy controls (HCs). After data preprocessed, amplitude of low frequency fluctuation (ALFF) of WM was calculated. ALFF values in different frequency bands were analyzed, including typical (0.01–0.15 Hz) band, slow-4 (0.027–0.073 Hz) and slow-5 (0.01–0.027 Hz) bands. In addition, the fractional anisotropy (FA) values in WM in 23 patients and 26 HCs were examined using tract-based spatial statistics (TBSS) and tractography based on diffusion tensor imaging (DTI). Pearson correlation analysis was applied to analyze the relationships between ALFF values and Hamilton Depression Scale (HAMD) and Hamilton Anxiety Scale (HAMA).

**Results:**

Compared with the HCs, MDD patients showed decreased ALFF values in posterior thalamic radiation (PTR) and superior longitudinal fasciculus (SLF) in slow-5 frequency band, no significant differences of ALFF values were found in typical and slow-4 frequency bands. In addition, there were no significant differences in FA values with TBSS analysis as well as the number of fibers in PTR and SLF with tractography analysis between two groups. Further correlation analysis showed that the ALFF value in SLF was negatively correlated with HAMA-2 score (*r* = −0.548, *p*_FDR_ = 0.037) in patients.

**Conclusion:**

Our results indicated that WM dysfunction may be associated with the pathophysiological mechanism of depression. Our study also suggested that the functional damage of the WM may precedes the structural damage in first-episode MDD patients. Furthermore, for mental disorders, slow-5 frequency band may be a more sensitive functional indicator for early detection of abnormal spontaneous brain activity in WM.

## 1. Introduction

Major depressive disorder (MDD) is highly prevalent worldwide with core symptoms of persistent sadness, worthlessness and guilt, often accompanied by other psychiatric symptoms such as cognitive deficits and anxiety, as well as some somatic symptoms including anorexia, bloating and muscle pain (1–3). However, our understanding of the pathophysiological basis of the disorder remains ambiguous. Using resting state functional MRI (rsfMRI), many studies focused on the functional alterations in brain to detect abnormal brain activities in different functional networks or neural pathways, which may imply the pathophysiological process of the MDD. But most studies mainly focused on the gray matter (GM), while ignoring the white matter (WM), which consists of myelinated axons that connect neurons in different regions of GM. According to a recent study, blood-oxygen-level-dependent (BOLD) signals in the WM represent brain activity and are detectable with traditional fMRI (4). It shows similar spectral and temporal characteristics in both GM and WM (5). These findings lend credence to the idea that changes in WM BOLD signals are bound up with tract-specific neural responses, which is inherent to WM or is a relay effect of GM.

A growing number of studies have proven that the structural and functional disruption of WM fibers may underlie the pathophysiology of MDD. Structurally, widespread WM abnormalities in MDD were revealed in a large number of diffusion tensor imaging (DTI) studies. Alterations in superior longitudinal fasciculus (SLF) (6, 7), corpus callosum (8), anterior limb of the internal capsule (9, 10), cingulum and anterior thalamic radiation (6) as well as posterior thalamic radiation (PTR) (11, 12) were found in some studies of the drug-naive first-episode MDD patients, which are associated with deficits in cognitive control, attentional processes and emotional awareness, consistent with the clinical manifestations such as grief, hopelessness and worthlessness, sluggish and inefficient. However, the results were variable and inconsistent in these studies.

In terms of WM function, few analyses have been focused on MDD. In recent studies reported that MDD patients exhibited reduced functional connectivity (FC) within WM, particularly in networks associated with cognitive and emotional functions (13, 14). For example, visual network and sensorimotor network, internal capsule, cingulum, and corpus callosum network showed reduced FC in drug-naive MDD patients compared with HCs. This illustrates that the networks involving in perception-motor system were severely damaged in MDD. Among psychiatric disorders other than depression, Yang et al. (15) found amplitude of low frequency fluctuation (ALFF) was lower in the splenium of corpus callosum in untreated schizophrenia patients compared with treated patients and controls. Right anterior corona radiata and cingulum were also observed reduced ALFF in schizophrenia patients who have not completely excluded medication (16). These abnormal tracts are associated with working memory, cognitive or perceptual dysfunction. In addition, there were the studies about abnormal spontaneous brain activity of WM in epilepsy, Parkinson’s and Alzheimer’s disease by ALFF (17–19). These studies suggest that ALFF is a sensitive indicator for studying brain WM spontaneous activity in neuropsychiatric disorders.

In 2007, Zang et al. (20) proposed for the first time an ALFF index defined by fluctuations of the BOLD signal at low frequency bands. The fMRI-based data-driven analysis technique can directly measure the ALFF of BOLD signals in each brain region, to reflect the intrinsic neuronal activity. In order to examine the relationship between neural activity and cognitive performance in various neurological disorders, researchers have recently begun to subdivide the frequency range of spontaneous brain activity (21, 22). Abnormal spontaneous neuronal activity can be obtained in many neuropsychiatric disorders by different specific frequency bands within the range of ALFF, including slow-4 (0.027–0.073 Hz) and slow-5 (0.01–0.027 Hz) bands. For example, in the sub-band study of autism spectrum disorders (23), predominant under connections between and within intrinsic connectivity networks in slow-5 frequency band; in postherpetic neuralgia, their intrinsic abnormal brain activity is associated with frequency, specifically bidirectional alterations of ALFF in slow-4 and slow-5 bands (24); as well as in amyotrophic lateral sclerosis, alterations in the strength of intrinsic functional connectivity were observed in some regions of the slow-4 and slow-5 bands (25). This implies that the dynamic changes in low-frequency fluctuations observed in neuropsychiatric disorders may be frequency dependent.

Tract-based spatial statistics (TBSS) is a relatively popular method for performing voxel-by-voxel DTI analysis, which sets a precedent by circumventing partial volume effects (PVE) by projecting volumetric data onto the WM skeleton (26). This method not only eliminates the need for data smoothing, but also alleviates the concerns of the voxel-based morphometry (VBM) framework previously used in many DTI studies (27). In addition, to assess the integrity of WM microstructure, the fractional anisotropy (FA) based on TBSS is a common and sensitive method for diffusion measurement (28). Because of the ability of FA (which indirectly reflects membrane integrity and myelin thickness), its reduction can indicate impairment and degeneration of WM fiber tracts (29). Tractography, as another focused technique, allows researchers to identify specific tracts by identifying seed ROIs (regions of interest) and estimating the number of tracts extending out from these ROIs. The benefit of the method is that WM indices along specific tracts can be examined rather than discrete parts of the brain as assessed using the ROIs and TBSS methods (30).

Therefore, using ALFF, TBSS, and tractography analyses, the purpose of this study is to reveal the changes in spontaneous brain activity and microstructure in WM in drug-naive first-episode MDD patients. We hypothesized that there were functional and structural alterations in WM which were related to the underlaying pathophysiological mechanisms in MDD, meanwhile, there were difference in sensitivity to detect the abnormal spontaneous neuronal activity in WM in different frequency bands when using ALFF as an indicator.

## 2. Materials and methods

### 2.1. Recruitment and qualification criteria of subjects

A total of 33 drug-naive first-episode MDD patients were recruited from the Psychiatry Department, First Affiliated Hospital, Zhejiang University School of Medicine. A consistent diagnosis was determined by two qualified psychiatrists. The criteria included were as follows: (1) satisfied the diagnostic criteria for depression in both the International Classification of Disease (ICD-10) and the Diagnostic and Statistical Manual of Mental Disorders (DSM-5); (2) this was the first episode and had not been treated with medication; (3) right-handed; (4) (17-item) Hamilton Depression Rating Scale (HAMD) score ≥17 points.

Thirty-four age- and gender -matched healthy controls (HCs) were recruited from hospital staff and surrounding community members at the First Affiliated Hospital of Zhejiang University School of Medicine. The non-patient version of the DSM-5 Structured Clinical Interview was used to exclude the HCs for any history of mental or neurological disorders.

The following excluded criteria applied to all subjects: (1) a background of severe organic brain illness and mental retardation; (2) alcohol, drug, and other abuse; (3) pregnant and menstruating women; (4) head motion >1.5 mm or 1.5°; (5) contraindications for magnetic resonance imaging (MRI), including claustrophobia and metallic implants.

### 2.2. Image acquisition

Magnetic resonance imaging images were captured with the same 3.0T GE Discovery MRI scanner (General Electric Medical Systems, Waukesha, WI, USA). All subjects were told to relax with eyes closed, but without falling asleep or having systematic thought during the scan. The head’s ability to move was limited by a foam pad.

The T1-weighted images were acquired on the Bravo sequence. The main scanning parameters were as follows: repetition time (TR) = 8.208 msec, echo time (TE) = 3.2 msec, field of view (FOV) = 256 mm × 256 mm^2^, flip angle = 8°, matrix = 256 × 256, slice thickness/spacing = 1 mm/0 mm, scanning time = 292 s.

The resting-state functional images were acquired by using gradient-echo imaging sequence. The main scanning parameters were as follows: 43 slices, TR = 2,000 msec, TE = 30 msec, FOV = 220 mm × 220 mm^2^, flip angle = 90°, matrix = 64 × 64, slice thickness/spacing = 3.2 mm/0.4 mm, scanning time = 400 s.

Diffusion tensor imaging images were acquired by using single-shot imaging sequence: TR = 8,600 msec, TE = 30 msec, FOV = 200 × 200 mm^2^, slice thickness = 2.4 mm (no slice gap), matrix = 128 × 128. Diffusion-sensitized gradients along 30 non-collinear directions were applied with the reference image.

### 2.3. Data preprocessing

#### 2.3.1. rsfMRI data preprocessing

Data Processing & Analysis for Brain Imaging (DPABI) v4.0^1^ (31) based on SPM12,^2^ on Matlab2017b,^3^ was used to preprocess rsfMRI data. The preprocessing includes the following steps: (1) remove the first 10 time points; (2) slice timing correction and realignment; (3) registration of T1-weighted image to the mean function image; (4) GM, WM, cerebrospinal fluid (CSF), skull, soft tissue, and extracerebral were segmented from the T1 images; (5) linear trend removed to correct for signal drift; (6) the 24 motion parameters (32), mean CSF signals and scrubbing regressors were regressed, to remove the influence of head motion and physiological noise; (7) spatially smooth GM and WM with a 4 mm full width half maximum isotropic Gaussian kernel; (8) normalized to the standard MNI template and resampled using the DARTEL algorithm to 3 × 3 × 3 mm^3^ voxels; (9) at the group level, create the WM mask for each subject based on the T1 images for statistical analysis.

#### 2.3.2. DTI data preprocessing

Diffusion tensor imaging preprocessing was performed using the FMRIB Software Library (FSL) version 6.0 (Oxford Centre for Functional MRI of the Brain, UK^4^). First, using the FSL brain extraction tool, the corrected images were subjected to skull stripping to eliminate non-brain tissue. The FSL Eddy Correction Tool was then used to fix the original diffusion-weighted pictures’ eddy current distortions and motion artifacts. Correct DTI data to create FA maps from feature values using tensor model. At last, these preprocessed data were subjected to TBSS and tractography analyses.

### 2.4. Analysis of ALFF, TBSS, and tractography

#### 2.4.1. Analysis of ALFF

Amplitude of low frequency fluctuation was calculated based on Fast Fourier transform and changed the time series into frequency domain at each voxel. First, get the square root of each power spectrum frequency, including the typical band (0.01–0.15 Hz), the slow-4 band (0.027–0.073 Hz), and the slow-5 band (0.01–0.027 Hz). The ALFF maps were calculated and normalized to Z scores for each subject. Second, two-sample *t*-test was carried out within the mask of group level WM for zALFF values in each frequency for subjects in both groups, respectively, MDD group with HCs group, two-tailed, with gender, age, and education levels as regression covariates. Finally, the *t*-test plots were corrected for multiple comparisons with multiple comparison correction for GRF, voxel *p* < 0.05, cluster *p* < 0.05.

#### 2.4.2. Analysis of TBSS

Fractional anisotropy images were non-linearly aligned to Montreal Neurological Institute-152 (MNI152) space using the FSL registration tool FMRIB58_FA_1mm. The mean FA image was obtained and refined to create the mean FA skeleton, which represents the center of all regions common to all objects. The FA threshold was set to be FA = 0.20. Project each individual FA map onto the skeleton. Statistical analysis was performed between two groups using gender, age, and education level as covariates in the regression. The significance threshold for TBSS was set to *p* < 0.05 and was corrected for multiple comparisons across voxels using the no-threshold clustering enhancement option, and results were inflated to output clusters.

#### 2.4.3. Analysis of tractography

(1) Diffusion tensor was calculated, eigenvector and eigenvalue maps as well as FA maps were constructed; (2) Fiber tacks were determined using the fiber assignment by continuous tracking method, with termination angle of 45° and FA greater than 0.2; (3) Reconstructed tracts were smoothed; (4) FA maps were registered to the FMRIB58-FA template corresponding to the MNI152 standard space using FSL’s linear image registration tool (FLIRT); (5) We focused on the white matter regions, the left PTR and SLF that were known significant difference in ALFF analysis, which were already in MNI152 standard space. The number of fibers obtained from the mask for the ROIs; (6) The number of fibers in PTR and SLF were analyzed by covariance between groups, group and gender were set as fixed variables, age and education level as covariates.

### 2.5. Correlation analysis

Data analysis was performed using Statistical Package for the Social Sciences (SPSS) 25.0 software. Student’s *t*-tests and Pearson Chi-square (χ^2^) tests were employed to assess differences in demographic and clinical characteristics between MDD and HCs. Two-sample *t*-tests were employed to identify differences in ALFF values between the MDD and HCs. Pearson correlation analysis was applied to analyze relationships between ALFF values and clinical characteristics including Hamilton Depression Scale (HAMD), Hamilton Anxiety Scale (HAMA) in MDD. The *p*-values were adjusted by using False Discovery Rated (FDR) correction. The above results were statistically significant at the *p* < 0.05 level.

## 3. Results

### 3.1. Subjects’ clinical and demographic characteristics

There were no significant differences in age (*p* = 0.071) and gender (*p* = 0.614), except for educational levels (*p* = 0.000), between MDD and HCs (Table 1). Compared with HCs, MDD group exhibited lower education levels. Given that learning and other life experiences are critical in shaping the functional and structural organization of the brain, and WM in the human brain undergoes a long period of maturation continues into adulthood (33, 34), age, and education levels were included as covariates in the regression. In addition, gender was also included as a covariate in order to eliminate its effect.

**TABLE 1:** Demographic data of the Subjects.

Variables	MDD group	HC group	*t*/*x*^2^ value	*P-*value
Count	33	34		
Age (years)	25.61 ± 6.442	28.85 ± 7.974	-1.836	0.071^a^
Gender (f/m)	26/7	25/9	0.255	0.614^b^
Education years	13.00 ± 2.915	16.85 ± 2.721	-4.138	0.000^a^
First episode/recurrent	33/0			
On medication/not	0/33			
Illness duration (months)	16.76 ± 17.586			
HAMD	23.27 ± 3.867			
HAMA	17.42 ± 5.414			

HAMD, Hamilton Depression Scale; HAMA, Hamilton Anxiety Scale.

^a^Student’s *t*-test.

^b^Pearson chi-square *t*-test.

### 3.2. Functional alterations of WM in ALFF

**FIGURE 1:**
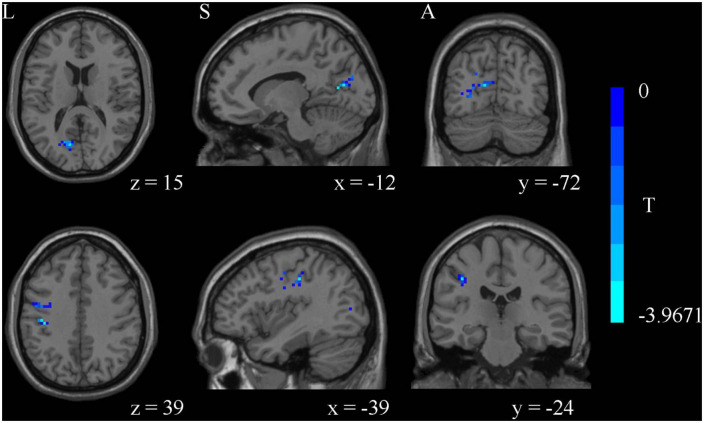
The amplitude of low frequency fluctuation (ALFF) analysis identified significantly decreased ALFF values of posterior thalamic radiation (PTR) and superior longitudinal fasciculus (SLF) in slow-5 band in major depressive disorder (MDD) compared to healthy controls (HCs).

**FIGURE 2:**
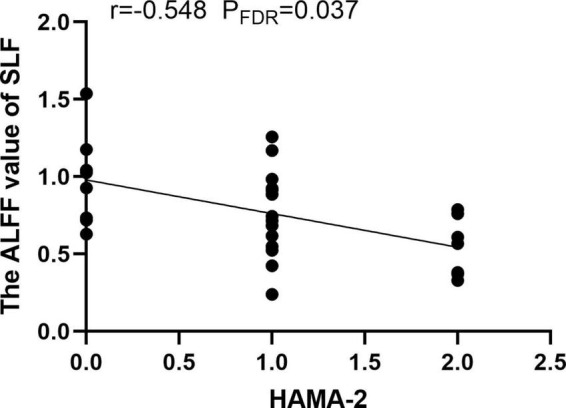
The amplitude of low frequency fluctuation (ALFF) value of superior longitudinal fasciculus (SLF) was negatively correlated with the HAMA-2 score.

Compared with the HCs, MDD patients showed significantly decreased ALFF values of PTR and SLF in slow-5 band, while no significant differences were found in the typical and slow-4 bands (Table 2 and Figure 1). Correlation analysis showed that the ALFF value of SLF was negatively correlated with HAMA-2 score (*r* = −0.548, *p*_FDR_ = 0.037) (Figure 2). No significant statistical results were found (*p* > 0.05) between the ALFF value of PTR and HAMD/HAMA scores.

**TABLE 2:** Results of two-sample *t*-test from amplitude of low frequency fluctuation (ALFF) analysis comparing major depressive disorder (MDD) and healthy controls (HCs).

Tract	Cluster size (voxels)	MNI coordinates			*T-*value
		***x***	***y***	***z***	
Typical band (0.01–0.15 Hz) -					
Slow-5 band (0.01–0.027 Hz) Posterior_thalamic_ radiation_L	70	−12	−72	15	−3.8159
Superior_longitudinal_ fasciculus_L	67	−39	−24	39	−3.9671
Slow-4 band (0.027–0.073 Hz) -					

The statistical threshold was set at voxel with *p* < 0.05 and cluster with *p* < 0.05 for GRF (Gaussian random field) corrected.

### 3.3. Structural alterations of WM in TBSS and tractography

**FIGURE 3:**
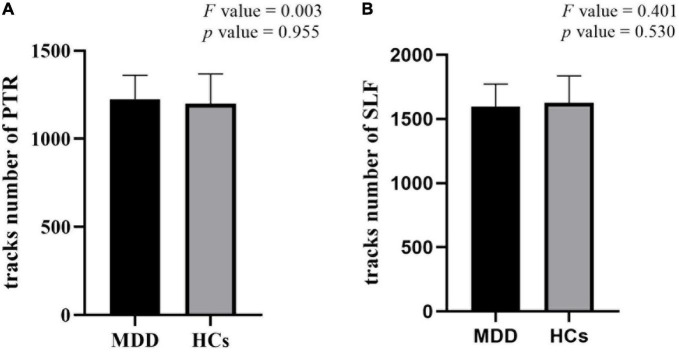
There were no significant differences in the number of fibers in posterior thalamic radiation (PTR) **(A)** and superior longitudinal fasciculus (SLF) **(B)** in tractography between major depressive disorder (MDD) and healthy controls (HCs).

There were no significant differences in FA values with TBSS analysis as well as the number of fibers in PTR and SLF with tractography analysis between MDD and HCs (Figure 3).

## 4. Discussion

It is well known that WM, which is enriched in myelinated axons, has an important position in the central nervous system, and therefore and therefore WM development is an extremely complex process regulated by a variety of intrinsic and extrinsic signals (35, 36). To rule out multiple episodes or potential confounding effects of medication, WM activity in drug-naive first-episode MDD patients was investigated. The patients showed decreased ALFF values in PTR and SLF in the slow-5 band, which mainly located in the subcortical WM regions. It has been proposed that the “disconnection syndrome” between cortical and subcortical regions in MDD is caused by the WM alterations of cortical-subcortical networks (37), which was consistent with our results. However, no significant differences were found in typical and slow-4 frequency bands as well as TBSS analysis between two groups. Our results indicated that the functional changes in WM may predate structural damage in the early stage of depression, in addition, slow-5 band may be a sensitive indicator to detect abnormal spontaneous activity in WM, which is similar to the dominance of slow-5 band in the study of intrinsic connectivity network in autism spectrum disorders (23).

The PTR is the WM fiber that connects the thalamus which is involved in processing and integrating sensory information enters the brain (38) to posterior visual cortex in the occipital lobe, including the optic radiation from the lateral geniculate body. The thalamus as a complex sensory information node (39) is a component of the emotion salience and regulation network and the cognitive/executive network (40). The abnormal function of the thalamus as one of the serotonergic nerve terminals may be related to the serotonergic mechanism of depression (41). Therefore, structural and functional abnormalities of the thalamus may be related to the pathological process of MDD, which can be considered as a potential marker. Several recent studies have shown that MDD patients have reduced thalamic volume (39, 42) and exhibit abnormal thalamic activation during emotional processing tasks (43). Additionally, abnormalities in the occipital lobe, which is another GM connected to the PTR, may also be associated with MDD. Because of its responsibility for visual perception and processing, reduced attention in MDD patients may be associated with attenuated occipital lobe function and patients with affective disorders were more likely to have atrophy of the occipital cortex (44). There were other studies of MDD showing reduced homogeneity of occipital lobe in rsfMRI (45) and reduced gray matter volume of occipital lobe in voxel-based morphometry analysis (46). The occipital cortex can also process non-visual information, such as tactile and auditory inputs, as well as some complex cognitive and speech related inputs (47–49). It has been shown that PTR is associated with deficits in visual construction, delayed memory capacity, cognitive control, attentional processes, and emotional awareness (50, 51). In our study, the decreased ALFF value of PTR indicated reduced spontaneous brain activity in the WM tract connecting the thalamus and occipital cortex, which may be due to impaired transmission in the PTR itself or secondary to structural damage or hypofunction in the thalamus and occipital lobes. Our results were in favor to the latter. Conversely, abnormalities in the WM may also lead to structural and functional changes in the associated GM, to trace up the source further studies are needed.

In this study, no significant statistical results were found between the ALFF value of PTR and HAMD/HAMA scores. Structural abnormalities in the PTR of MDD patients have been reported in previous studies (11, 12, 52), but not shown in our study. These inconsistent results may be due to different patient conditions. Compared with their studies, the patients were mild depressive symptoms, shorter course of disease and all were the drug-naive first-episode in our study. Our results suggested that the structure of the WM is not yet altered in the early stages of the MDD.

The SLF is considered to be the largest associative fiber bundle system in the brain (53), which lays connections between the frontal and parietal lobe (54). It is considered as a higher-order multisensory associative system, which has been commonly reported to be associated with somatic discomfort (55), working memory performance (56, 57), attention (58, 59), executive function, and emotion (60). The fiber bundle is composed of three distinct bundles including SLF-I (dorsal pathway), SLF-II (middle pathway), and SLF-III (ventral pathway) (61). The SLF-I travels within the cingulate or paracingulate gyrus, connecting the anterior cingulate cortex, the superior frontal gyrus, the precentral gyrus, the central paracentral lobule, and the precuneus (62, 63). The reduced ALFF region in SLF in our study was located near the pre/postcentral gyrus at SLF-I. Correlation analysis showed that the ALFF value of SLF was negatively correlated with HAMA-2 (tension) score, suggesting that impaired function in SLF-I was severe with the increase of patients’ tension. Previous study suggested that SLF may be more severely impaired in patients with anxious depression than in non-anxious patients, which may lead to impaired cognitive and emotional functioning and correspondingly more severe symptoms (64). In addition, the study also showed that a considerable number of MDD patients have somatic symptoms such as exhaustion and gastrointestinal discomfort, these can result from abnormalities in the pre/postcentral gyrus, which are located in the brain’s somatomotor and somatosensory regions and are responsible for integrating and processing bodily information (55). The regional homogeneity and ALFF values significantly decreased in the bilateral precentral gyrus, bilateral postcentral gyrus, and left paracentral gyrus were found in the somatic MDD group compared to the pure depression group (65). Similarly, our uncorrected correlation analysis result showed a negative correlation trend (*r* = −0.387, *p* = 0.034) between the ALFF values of SLF-I and the HAMD-17 anxiety/somatization factor items in patients, which may suggested more severe dysfunction in SLF-I with the increase of patients’ somatic symptoms. Therefore, in this study, we speculate that decreased ALFF values in SLF-I are likely to be secondary to structural or functional impairment of the pre/postcentral gyrus.

In drug-naive MDD patients, recent studies found that the decreased FA in the SLF by TBSS analysis was significantly correlated with decreased fractional amplitude of low-frequency fluctuation in left pre/postcentral gyrus, this simultaneous disruption of structure and function may be associated with impaired cognitive and emotional functioning in MDD patients (7). However, the difference of FA in SLF was not found in our TBSS analysis between two groups. We speculate that it may be due to the higher HAMD scores, the longer duration of illness and the mixed recurrent patients of MDD in Zeng’s study. Previous study has concluded that lower FA values in the SLF were related with more hospitalizations and that SLF fiber integrity may reflect the cumulative disease burden in MDD (66). It also suggested that the damage in WM structures did not occur in the early stage of the disease, but emerged as the disease progresses further. Therefore, our results suggested that the BOLD signal changes of SLF were likely related to the inherent tract-specific neural response of WM or the relay effect of the corresponding GM. Of course, abnormalities in the WM may also lead to structural and functional changes in the associated GM, to trace up the source more in-depth research is needed.

Although, the significance and origin of the signals from different frequency bands are unclear, it has been proposed that large neuronal systems’ integration and long-distance connections have been linked to low-frequency oscillations (67) and different lower frequency bands are associated with different neural processes and physiological functions (21, 67). Slow-5 band maybe a more sensitive frequency to the activity of cortical neurons, and the oscillations of basal ganglia are stronger in slow-4 band (17, 22, 67). Previous rsfMRI study (17) also showed that the local separation characteristics of the slow-5 band are always greater than those of the slow-4 band, the slow-4 band component in the typical band may mask the characteristics of the slow-5 band component, thus reducing the sensitivity of the classical band. Our study also illustrates that slow-5 band may be a more sensitive functional indicator for revealing the spontaneous brain activity in WM than the typical and slow 4-bands in the early stage of the disorder.

At last, our study still has several limitations that need to be considered. First, the sample size was relatively small, further studies with larger samples are needed. Second, as a result of using a cross-sectional design, the present study was unable to determine whether progressive pathological changes occurred in the WM of MDD patients, further longitudinal studies would be helpful. Last, our analysis was limited to the WM, to trace up the source of abnormal WM function in further studies could be extended to assess potential GM alterations.

## 5. Conclusion

Our results indicated that abnormal WM spontaneous activity may be one of the pathophysiological mechanisms in MDD, which is associated with cognitive and emotional dysfunction and somatic discomfort in patients. Our study also suggested, in the early stage of disorders, that functional damage to WM may precede structural damage. Furthermore, slow-5 frequency band may be a more sensitive functional indicator for early detection of abnormal spontaneous brain activity in WM.

## Data availability statement

The raw data supporting the conclusions of this article will be made available by the authors, without undue reservation.

## Ethics statement

The studies involving human participants were reviewed and approved by the Ethics Committee of The First Affiliated Hospital, Zhejiang University School of Medicine. Written informed consent to participate in this study was provided by the participants’ legal guardian/next of kin.

## Author contributions

QG collected the samples and wrote the initial manuscript. JD, SC, and TC collected the samples, supervised the data, and conducted quality control. JZ analyzed the data. HY supervised, designed the research, collected the samples, and revised the manuscript. All authors reviewed and approved the submitted manuscript.

## Footnotes

1
http://rfmri.org/dpabi

2
www.fil.ion.ucl.ac.uk/spm

3
ww2.mathworks.cn/products/matlab.html

4
http://www.fmrib.ox.ac.uk/fsl

## References

[1] Jia Z Huang X Wu Q Zhang T Lui S Zhang J High-field magnetic resonance imaging of suicidality in patients with major depressive disorder. *Am J Psychiatry.* (2010). 167:1381–90. 10.1176/appi.ajp.2010.09101513 20843871

[2] Chen T Kendrick K Wang J Wu M Li K Huang X Anomalous single-subject based morphological cortical networks in drug-naive, first-episode major depressive disorder. *Hum Brain Mapp.* (2017) 38:2482–94. 10.1002/hbm.23534 28176413 PMC6866860

[3] Gong Q He Y. Depression, neuroimaging and connectomics: a selective overview. *Biol Psychiatry.* (2015) 77:223–35. 10.1016/j.biopsych.2014.08.009 25444171

[4] Gore J Li M Gao Y Wu T Schilling K Huang Y Functional MRI and resting state connectivity in white matter - a mini-review. *Magn Reson Imaging.* (2019) 63:1–11. 10.1016/j.mri.2019.07.01 31376477 PMC6861686

[5] Ding Z Newton A Xu R Anderson A Morgan V Gore J. Spatio-temporal correlation tensors reveal functional structure in human brain. *PLoS One.* (2013) 8:e82107. 10.1371/journal.pone.0082107 24339997 PMC3855380

[6] Yang X Wang Y Wang D Wang D Tian K Cheung E White matter microstructural abnormalities and their association with anticipatory anhedonia in depression. *Psychiatry Res Neuroimaging.* (2017) 264:29–34. 10.1016/j.pscychresns.2017.04.005 28437669

[7] Zeng M Yu M Qi G Zhang S Ma J Hu Q Concurrent alterations of white matter microstructure and functional activities in medication-free major depressive disorder. *Brain Imaging Behav.* (2021) 15:2159–67. 10.1007/s11682-020-00411-6 33155171

[8] Han K Choi S Jung J Na K Yoon H Lee M Cortical thickness, cortical and subcortical volume, and white matter integrity in patients with their first episode of major depression. *J Affect Disord.* (2014) 155:42–8. 10.1016/j.jad.2013.10.021 24210630

[9] Zhu X Wang X Xiao J Zhong M Liao J Yao S. Altered white matter integrity in first-episode, treatment-naive young adults with major depressive disorder: a tract-based spatial statistics study. *Brain Res.* (2011) 1369:223–9. 10.1016/j.brainres.2010.10.104 21047498

[10] Chen G Hu X Li L Huang X Lui S Kuang W Disorganization of white matter architecture in major depressive disorder: a meta-analysis of diffusion tensor imaging with tract-based spatial statistics. *Sci Rep.* (2016) 6:21825. 10.1038/srep21825 26906716 PMC4764827

[11] Bessette K Nave A Caprihan A Stevens M. White matter abnormalities in adolescents with major depressive disorder. *Brain Imaging Behav.* (2014) 8:531–41. 10.1007/s11682-013-9274-8 24242685

[12] Liao Y Huang X Wu Q Yang C Kuang W Du M Is depression a disconnection syndrome? Meta-analysis of diffusion tensor imaging studies in patients with MDD. *J Psychiatry Neurosci.* (2013) 38:49–56. 10.1503/jpn.110180 22691300 PMC3529219

[13] Zhang Y Kong Y Liu X Gao H Yin Y Hou Z Desynchronized functional activities between brain white and gray matter in major depression disorder. *J Magn Reson Imaging.* (2021) 53:1375–86. 10.1002/jmri.27466 33305508

[14] Zhao Y Zhang F Zhang W Chen L Chen Z Lui S Decoupling of gray and white matter functional networks in medication-naïve patients with major depressive disorder. *J Magn Reson Imaging.* (2021) 53:742–52. 10.1002/jmri.27392 33043540

[15] Yang C Zhang W Yao L Shah C Zeng J Yang Z Functional alterations of white matter in chronic never-treated and treated schizophrenia patients. *J Magn Reson Imaging.* (2020) 52:752–63. 10.1002/jmri.27028 31859423

[16] Liu N Lencer R Yang Z Zhang W Yang C Zeng J Altered functional synchrony between gray and white matter as a novel indicator of brain system dysconnectivity in schizophrenia. *Psychol Med.* (2021) 52:2540–8. 10.1017/s0033291720004420 33436114

[17] Xue S Li D Weng X Northoff G Li D. Different neural manifestations of two slow frequency bands in resting functional magnetic resonance imaging:a systemic survey at regional, interregional, and network levels. *Brain Connect.* (2014) 4:242–55. 10.1089/brain.2013.0182 24456196

[18] Caso F Agosta F Mattavelli D Migliaccio R Canu E Magnani G White matter degeneration in atypical alzheimer disease. *Radiology.* (2015) 277:162–72. 10.1148/radiol.2015142766 26018810

[19] Dong D Wang Y Chang X Jiang Y Klugah-Brown B Luo C Shared abnormality of white matter integrity in schizophrenia and bipolar disorder: a comparative voxel-based meta-analysis. *Schizophr Res.* (2017) 185:41–50. 10.1016/j.schres.2017.01.005 28082140

[20] Zang Y He Y Zhu C Cao Q Sui M Liang M Altered baseline brain activity in children with ADHD revealed by resting-state functional MRI. *Brain Dev.* (2007) 29:83–91. 10.1016/j.braindev.2006.07.002 16919409

[21] Knyazev G. Motivation, emotion, and their inhibitory control mirrored in brain oscillations. *Neurosci Biobehav Rev.* (2007) 31:377–95. 10.1016/j.neubiorev.2006.10.004 17145079

[22] Zuo X Di Martino A Kelly C Shehzad Z Gee D Klein D The oscillating brain: complex and reliable. *Neuroimage.* (2010) 49:1432. 10.1016/j.neuroimage.2009.09.037 19782143 PMC2856476

[23] Duan X Chen H He C Long Z Guo X Zhou Y Resting-state functional under-connectivity within and between large-scale cortical networks across three low-frequency bands in adolescents with autism. *Prog Neuropsychopharmacol Biol Psychiatry.* (2017) 79:434–41. 10.1016/j.pnpbp.2017.07.027 28779909

[24] Gu L Hong S Jiang J Liu J Cao X Huang Q Bidirectional alterations in ALFF across slow-5 and slow-4 frequencies in the brains of postherpetic neuralgia patients. *J Pain Res.* (2018) 12:39–47. 10.2147/JPR.S179077 30588078 PMC6302822

[25] Li F Zhou F Huang M Gong H Xu R. Frequency-specific abnormalities of intrinsic functional connectivity strength among patients with amyotrophic lateral sclerosis: a resting-state fMRI study. *Front Aging Neurosci.* (2017) 9:351. 10.3389/fnagi.2017.00351 29163133 PMC5681965

[26] Smith S Jenkinson M Johansen-Berg H Rueckert D Nichols T Mackay C Tract-based spatial statistics: voxelwise analysis of multi-subject diffusion data. *Neuroimage.* (2006) 31:1487–505. 10.1016/j.neuroimage.2006.02.024 16624579

[27] Jones D Symms M Cercignani M Howard R. The effect of filter size on VBM analyses of DT-MRI data. *Neuroimage.* (2005) 26:546–54. 10.1016/j.neuroimage.2005.02.013 15907311

[28] Cui Y Dong J Yang Y Yu H Li W Liu Y White matter microstructural differences across major depressive disorder, bipolar disorder and schizophrenia: a tract-based spatial statistics study. *J Affect Disord.* (2020) 260:281–6. 10.1016/j.jad.2019.09.029 31521864

[29] Beaulieu C. The basis of anisotropic water diffusion in the nervous system - a technical review. *Nmr Biomed.* (2002) 15:435–55. 10.1002/nbm.782 12489094

[30] Olvet D Delaparte L Yeh F DeLorenzo C McGrath P Weissman M A comprehensive examination of white matter tracts and connectometry in major depressive disorder. *Depress Anxiety.* (2016) 33:56–65. 10.1002/da.22445 26477532 PMC4701622

[31] Yan C Wang X Zuo X Zang Y. DPABI: data processing & analysis for (resting-state) brain imaging. *Neuroinformatics.* (2016) 14:339–51. 10.1007/s12021-016-9299-4 27075850

[32] Friston K Williams S Howard R Frackowiak R Turner R. Movement-related effects in fMRI time-series: movement artifacts in fMRI. *Magn Reson Med.* (1996) 35:346–55. 10.1002/mrm.191035 8699946

[33] Walhovd K Westlye L Amlien I Espeseth T Reinvang I Raz N Consistent neuroanatomical age-related volume differences across multiple samples. *Neurobiol Aging.* (2011) 32:916–32. 10.1016/j.neurobiolaging.2009.05.013 19570593 PMC4040218

[34] Lebel C Treit S Beaulieu C. A review of diffusion MRI of typical white matter development from early childhood to young adulthood. *NMR Biomed.* (2019) 32:e3778. 10.1002/nbm.3778 28886240

[35] Filley C Fields R. White matter and cognition: making the connection. *J Neurophysiol.* (2016) 116:2093–104. 10.1152/jn.00221.2016 27512019 PMC5102321

[36] Rice D Barone S Jr. Critical periods of vulnerability for the developing nervous system: evidence from humans and animal models Environ. *Health Perspect.* (2000) 108:511–3. 10.1289/ehp.00108s3511 10852851 PMC1637807

[37] Sexton C Mackay C Ebmeier K. A systematic review of diffusion tensor imaging studies in affective disorders. *Biol Psychiatry.* (2009) 66:814–23. 10.1016/j.biopsych.2009.05.024 19615671

[38] Clinton S Meador-Woodruff J. Thalamic dysfunction in schizophrenia: neurochemical, neuropathological, and in vivo imaging abnormalities. *Schizophr Res.* (2004) 69:237–53. 10.1016/j.schres.2003.09.017 15469196

[39] Nugent A Davis R Zarate C Drevets W. Reduced thalamic volumes in major depressive disorder. *Psychiatry Res.* (2013) 213:179–85. 10.1016/j.pscychresns.2013.05.004 23850106 PMC3756884

[40] Yamamura T Okamoto Y Okada G Takaishi Y Takamura M Mantani A Association of thalamic hyperactivity with treatment-resistant depression and poor response in early treatment for major depression: a resting-state fMRI study using fractional amplitude of low-frequency fluctuations. *Transl Psychiatry.* (2016) 6:e754. 10.1038/tp.2016.18 26954981 PMC4872444

[41] Rosa-Neto P Diksic M Okazawa H Leyton M Ghadirian N Mzengeza S. Measurement of brain regional alpha-(11C) methyl-L-tryptophan trapping as a measure of serotonin synthesis in medication-free patients with major depression. *Arch Gen Psychiatry.* (2004) 61:556–63. 10.1001/archpsyc.61.6.556 15184235

[42] Webb C Weber M Mundy E Killgore W. Reduced gray matter volume in the anterior cingulate, orbitofrontal cortex and thalamus as a function of mild depressive symptoms: a voxel-based morphometric analysis. *Psychol Med.* (2014) 44:2833–43. 10.1017/S0033291714000348 25066703 PMC4280261

[43] Miller C Hamilton J Sacchet M Gotlib I. Meta-analysis of functional neuroimaging of major depressive disorder in youth. *JAMA Psychiatry.* (2015) 72:1045–53. 10.1001/jamapsychiatry.2015.1376 26332700 PMC11890701

[44] Lai C Hsu Y. A subtle grey-matter increase in first-episode, drug-naive major depressive disorder with panic disorder after 6 weeks’ duloxetine therapy. *Int J Neuropsychopharmacol.* (2011) 14:225–35. 10.1017/S1461145710000829 20663271

[45] Peng D Jiang K Fang Y Xu Y Shen T Long X Decreased regional homogeneity in major depression as revealed by resting-state functional magnetic resonance imaging. *Chin Med J.* (2011) 124:369–73. 10.3760/cma.j.issn.0366-6999.2011.03.009 21362335

[46] Yang Y Li X Cui Y Liu K Qu H Lu Y Reduced gray matter volume in orbitofrontal cortex across schizophrenia, major depressive disorder, and bipolar disorder: a comparative imaging study. *Front Neurosci.* (2022) 16:919272. 10.3389/fnins.2022.919272 35757556 PMC9226907

[47] Cohen L Celnik P Pascual-Leone A Corwell B Falz L Dambrosia J Functional relevance of cross-modal plasticity in blind humans. *Nature.* (1997) 389:180–3. 10.1038/38278 9296495

[48] Renier L Anurova I de Volder A Carlson S VanMeter J Rauschecker J. Preserved functional specialization for spatial processing in the middle occipital gyrus of the early blind. *Neuron.* (2010) 68:138–48. 10.1016/j.neuron.2010.09.021 20920797 PMC2951740

[49] Bedny M Pascual-Leone A Dravida S Saxe R. A sensitive period for language in the visual cortex: distinct patterns of plasticity in congenitally versus late blind adults. *Brain Lang.* (2012) 122:162–70. 10.1016/j.bandl.2011.10.005 22154509 PMC3536016

[50] Chaddock-Heyman L Erickson K Voss M Powers J Knecht A Pontifex M White matter microstructure is associated with cognitive control in children. *Biol Psychol.* (2013) 94:109–15. 10.1016/j.biopsycho.2013.05.008 23714226 PMC3742734

[51] Killgore W Vanuk J Shane B Weber M Bajaj S. A randomized, double-blind, placebo-controlled trial of blue wavelength light exposure on sleep and recovery of brain structure, function, and cognition following mild traumatic brain injury. *Neurobiol Dis.* (2020) 134:104679. 10.1016/j.nbd.2019.104679 31751607

[52] Hermesdorf M Berger K Szentkirályi A Schwindt W Dannlowski U Wersching H. Reduced fractional anisotropy in patients with major depressive disorder and associations with vascular stiffness. *Neuroimage Clin.* (2017) 14:151–5. 10.1016/j.nicl.2017.01.013 28180073 PMC5279701

[53] Schmahmann J Pandya D Wang R Dai G D’Arceuil H de Crespigny A Association fibre pathways of the brain: parallel observations from diffusion spectrum imaging and autoradiography. *Brain.* (2007) 130:630–53. 10.1093/brain/awl359 17293361

[54] Caspers S Zilles K. Microarchitecture and connectivity of the parietal lobe. *Handb Clin Neurol.* (2018) 151:53–72. 10.1016/B978-0-444-63622-5.00003-6 29519479

[55] Zu M Wang A Bai T Xie W Guan J Tian Y Resting-state functional connectivity between centromedial amygdala and insula as related to somatic symptoms in depressed patients: a preliminary study. *Psychosom Med.* (2019) 81:434–40. 10.1097/psy.0000000000000697 31008903

[56] Vestergaard M Madsen K Baaré W Skimminge A Ejersbo L Ramsøy T White matter microstructure in superior longitudinal fasciculus associated with spatial working memory performance in children. *J Cogn Neurosci.* (2011) 23:2135–46. 10.1162/jocn.2010.21592 20964591

[57] Alagapan S Lustenberger C Hadar E Shin H Frhlich F. Low-frequency direct cortical stimulation of left superior frontal gyrus enhances working memory performance. *Neuroimage.* (2019) 184:697–706. 10.1016/j.neuroimage.2018.09.064 30268847 PMC6240347

[58] Klarborg B Skak Madsen K Vestergaard M Skimminge A Jernigan T Baaré W. Sustained attention is associated with right superior longitudinal fasciculus and superior parietal white matter microstructure in children. *Hum Brain Mapp.* (2013) 34:3216–32. 10.1002/hbm.22139 22806938 PMC6870398

[59] Urger S De Bellis M Hooper S Woolley D Chen S Provenzale J. The superior longitudinal fasciculus in typically developing children and adolescents: diffusion tensor imaging and neuropsychological correlates. *J Child Neurol.* (2015) 30:9–20. 10.1177/0883073813520503 24556549 PMC4138302

[60] Biesbroek JM Kuijf HJ van der Graaf Y Vincken KL Postma A Mali WP Association between subcortical vascular lesion location and cognition: a voxel-based and tract-based lesion-symptom mapping study. The SMART-MR study. *PLoS One.* (2013) 8:e60541. 10.1371/journal.pone.0060541 23593238 PMC3620525

[61] Martino J De Witt Hamer P Berger MT Lawton M Arnold C de Lucas E Analysis of the subcomponents and cortical terminations of the perisylvian superior longitudinal fasciculus: a fiber dissection and DTI tractography study. *Brain Struct Funct.* (2013) 218:105–21. 10.1007/s00429-012-0386-5 22422148

[62] Komaitis S Skandalakis G Kalyvas A Drosos E Lani E Emelifeonwu J Dorsal component of the superior longitudinal fasciculus revisited: novel insights from a focused fiber dissection study. *J Neurosurg.* (2019) 132:1265–78. 10.3171/2018.11.JNS182908 30835690

[63] Wang X Pathak S Stefaneanu L Yeh F Li S Fernandez-Miranda J. Subcomponents and connectivity of the superior longitudinal fasciculus in the human brain. *Brain Struct Funct.* (2016) 221:2075–92. 10.1007/s00429-015-1028-5 25782434

[64] Xia W Zhou R Zhao G Wang F Mao R Peng D Abnormal white matter integrity in Chinese young adults with first-episode medication-free anxious depression: a possible neurological biomarker of subtype major depressive disorder. *Neuropsychiatr Dis Treat.* (2018) 14:2017–26. 10.2147/NDT.S169583 30127612 PMC6091250

[65] Liu P Tu H Zhang A Yang C Liu Z Lei L Brain functional alterations in MDD patients with somatic symptoms: a resting-state fMRI study. *J Affect Disord.* (2021) 295:788–96. 10.1016/j.jad.2021.08.143 34517253

[66] Meinert S Leehr E Grotegerd D Repple J Förster K Winter N White matter fiber microstructure is associated with prior hospitalizations rather than acute symptomatology in major depressive disorder. *Psychol Med.* (2020) 52:1166–74. 10.1017/S0033291720002950 32921338

[67] Buzsáki G Draguhn A. Neuronal oscillations in cortical networks. *Science.* (2004) 304:1926–9. 10.1126/science.1099745 15218136

